# From protocolized to person-centered chronic care in general practice: study protocol of an action-based research project (COPILOT)

**DOI:** 10.1017/S1463423619000550

**Published:** 2019-09-24

**Authors:** Mieke J.L. Bogerd, Pauline Slottje, Francois G. Schellevis, Anneliet Giebels, Mieke Rijken, Hein P.J. van Hout, Marcel E. Reinders

**Affiliations:** 1 Amsterdam UMC, Vrije Universiteit Amsterdam, department of General Practice & Elderly Care Medicine, Amsterdam Public Health research institute, De Boelelaan 1117, Amsterdam, Netherlands; 2 NIVEL (Netherlands Institute for Health Services Research), Utrecht, the Netherlands; 3 Huisartsen Coöperatie Zuid Kennemerland (HCZK), Kleermakerstraat 51, Velserbroek, Netherlands

**Keywords:** action-based research, chronic diseases, general practice, multimorbidity, person-centered care, primary care, quadruple aim

## Abstract

**Aim:**

To develop a proactive person-centered care approach for persons with (multiple) chronic diseases in general practice, and to explore the impact on ‘Quadruple aims’: experiences of patients and professionals, patient outcomes and costs of resources use.

**Background:**

The management of people with multiple chronic diseases challenges health care systems designed around single disease. Patients with multimorbidity often receive highly fragmented care that may lead to inefficient, ineffective and potentially harmful treatments and neglect of essential health needs. A more comprehensive, person-centered approach is advocated for persons with multiple morbidities. However, examples on how to provide more person-centered care and evidence of its impact are scarce. A group of Dutch general practitioners (GPs) took the initiative to develop such a care approach.

**Methods/Design:**

Mixed methods with a development and pilot-testing phase. The proactive person-centered approach will be developed using an action-based research design consisting of multiple plan-act-observe-reflect-adjust cycles. In each cycle, experiences of patients and primary care professionals from 13 practices will be collected via interviews, observations and focus groups. Starting point for the first cycle is a ‘person-centered consultation’ of up to 1 h in which the GP discusses the health status and health care needs of the patient. Furthermore, shared decisions between GP and patient are made on treatment goals and follow-up. In the pilot-test phase, a nested case cohort study allows to explore the impact of the new approach on ‘Quadruple aim’ outcomes comparing persons with and without exposure to the new care approach.

**Discussion:**

This study will provide a proactive person-centered approach for persons with multimorbidity in primary care and estimate its potential impact on quadruple outcomes.

## Introduction

Of people living with chronic diseases, 52% is multimorbid. Multimorbidity can be defined as ‘the co-existence of two or more chronic conditions in one person’ (van den Akker *et al*., 1996: 69) and its prevalence increases with age, affecting more than 66% of people aged 65 or older (Hilderink and Verschuuren, 2018). Multimorbidity can have a major impact on patients’ lives in both younger and older adults (Van Merode *et al*., 2018). Compared to patients with a single chronic disease, patients with multimorbidity have a lower life expectancy, are more likely to be admitted to a hospital, have a poorer quality of life and are at increased risk of polypharmacy-associated adverse drug events and difficulties with adherence (Boyd *et al*., 2005; Onder *et al*., 2015). Moreover, patients with multimorbidity usually require long-term multidisciplinary care. At a societal level, multimorbidity is associated with high health care costs (Hopman *et al*., 2015). In the United States, people with multimorbidity account for more than two-thirds of total health care spending (Salisbury *et al*., 2018).

In the Netherlands, care for patients with three highly frequent chronic diseases in the primary care setting is organized in structured disease management programs: diabetes mellitus, chronic obstructive pulmonary disease (COPD) and cardiovascular diseases (cardiovascular risk management, CVRM). These programs are highly protocolized and have a strong disease-specific orientation. Such disease management may neglect essential aspects for patients with multimorbidity such as daily and social functioning, goals in life, care preferences, as well as cognitive and emotional status (Wagner *et al*., 2001; Onder *et al*., 2015). Furthermore, recommendations based on disease-specific guidelines can also be inappropriate for patients with coexisting conditions due to contradictory or complex medication and lifestyle regimes (Boyd *et al*., 2005; Salisbury *et al*., 2018). As a result, multimorbid patients often receive untailored, highly fragmented care that may lead to incomplete, inefficient, ineffective and potentially harmful treatments (Boyd *et al*., 2005; Heide *et al*., 2018). In addition, these strongly protocolized working methods led to a high-experienced (unnecessary) regulatory and administrative burden by primary care professionals (Landelijke Huisartsen Vereniging (LHV), 2018).

To improve the quality of care for patients with multimorbidity while limiting the regulatory burden for care professionals, a shift from the currently protocolized disease-specific care to a more comprehensive and person-centered chronic care is proposed (Palmer *et al*., 2018). However, the evidence on how to manage multiple chronic diseases in a more person-centered way is limited yet.

Hopman *et al*. published a systematic review on the effectiveness of innovative care models for complex patients such as frail elderly and persons with multimorbidity. Nineteen studies from America, Australia, Canada and the Netherlands were included. Impact was assessed with a wide range of outcome measures, for example, patient outcomes including patient satisfaction, health-related quality of life, depressive symptoms, functional status and mortality, as well as health care utilization and costs.

Hopman *et al*. reported that integrated (non-fragmented) care models have positive effects on the quality of care and quality of life experienced by frail patients and patients with multimorbidity (Hopman *et al*., 2016). No impact was found on health care costs (Hopman *et al*., 2016). Smith *et al*. reported a systematic review of interventions in primary care and community settings. Organizational interventions for patients with multimorbidity, which focused on specific risk factor management or target areas where patients have difficulties, such as with functional ability, are more likely to be effective than other types of organizational interventions (Smith *et al*., 2012). Furthermore, Salisbury *et al*. recently reported a large cluster-randomized controlled trial (RCT) in general practice including a patient-centered care arm which improved patient experiences compared to usual care. It did not affect quality of life or the burden of illness or treatment (Salisbury *et al*., 2018).

Recent insights comment that person-centeredness is not well captured by conventional outcome assessments which often concern disease-specific indicators, such as HbA1c levels, hypertension control or generic measures such as quality of life or mortality (Reuben and Tinetti, 2012). Alternative approaches focus more on patient’s individual health goals throughout an intervention which may be related to a variety of domains, including functional ability or social activities. However, since existing literature on the content, effectiveness and appropriate outcomes to evaluate the effectiveness of innovative chronic primary care models for patients with complex care needs is scarce, more research on this topic is needed.

The study described in this development paper, named ‘COPILOT’, is an initiative of a group of Dutch GPs joined in ‘Huisartsen Coöperatie Zuid-Kennemerland’ (HCZK), who want to provide proactive and person-centered care for their patients with chronic diseases. This bottom-up approach emphasizes even more the urgent need for a new approach. The first aim of this study is to develop a new chronic primary care approach suitable for patients with multiple chronic diseases. The second aim of the pilot is to explore this new approach on achieving the ‘Quadruple aim’ outcomes (Bodenheimer and Sinsky, 2014). The ‘Quadruple aims’ comprise four dimensions:

- Enhancing patient experience
- Improving patients’ health
- Improving the work experiences of health care professionals
- Reducing societal costs and costs of resource use

## Methods

### *Study design*

The study uses mixed qualitative and quantitative methods in a development phase and a pilot-test phase. During the development phase, a new approach for chronic care in the primary setting will be developed using an action-based research design. During the test phase, the impact of this new approach for primary chronic care on the ‘Quadruple aim’ outcomes will be explored in a nested cohort study comparing persons with and without exposure to the new approach.

#### Development phase

In the development phase, an action-based research design will be applied. Action-based research is a type of emancipatory research in which research participants (in this study GPs, patients and researchers) are involved in the process of decision-making (Cordeiro *et al*., 2015).

**Figure 1.**
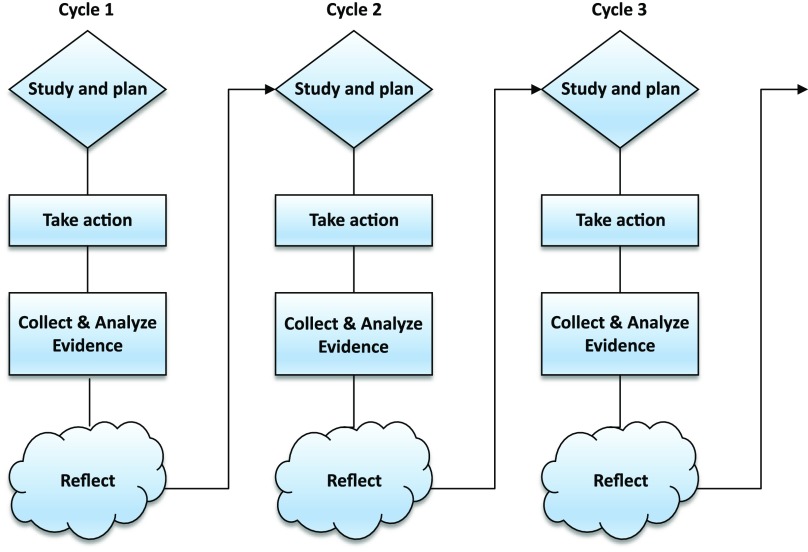
Multiple plan-act-observe-reflect-adjust cycles

The development phase consists of multiple plan-act-observe-reflect-adjust cycles (Figure 1) of approximately six months (Cordeiro *et al*., 2015). A cycle can be divided into an ‘action phase’ of four months and an ‘evaluation and adjust phase’ of two months. During the action phase, GPs and patients are the ‘actors’ and data will be collected. During the evaluation phase, all research participants reflect upon this action phase. During every cycle, the experiences of patients and GPs are collected through interviews, focus groups as well as questionnaires. Researchers process this information through thematic interpretation of both the qualitative interview material and inspection of the quantitative material. They identify barriers, facilitators and potential improvements. Prior to the next cycle, this information is shared with and discussed among the participating GPs. This GP group is invited to propose and decide upon adjustments, which will then be tested in the next cycle. When (substantial) adjustments are no longer needed, the development phase will be complete. We expect to reach this saturation after three cycles.

#### Chronic care approach: ‘person-centered consultation’

**Figure 2.**
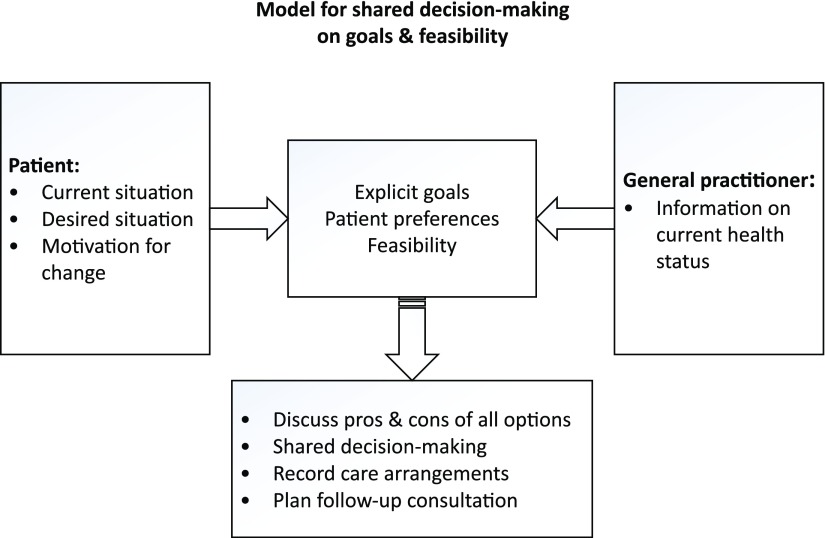
Model used for person-centered consulation (NHG)

Prior to the first research cycle, GPs who initiated the change in care delivery for chronic patients with multimorbidity organized multiple brainstorm sessions to discuss potential ideas for a new chronic care approach. As starting point for the first cycle, the GPs decided to preselect patients and to start a so-called ‘person-centered consultation’ which may take up to an hour instead of the regular 10-min consultation. During this consultation, the health status and health care needs of the patient will be discussed. A so-called ‘pre-pilot’ was conducted by 8 GPs who together performed 24 ‘person-centered consultations’ during a time period of three months. Based on their experiences, GPs expect that increased understanding of a patient’s context will positively affect the quality of provided care. The Dutch College of General Practitioners (NHG) encourages the use of a model for shared decision-making on goals and care arrangements (Figure 2) (NHG, 2017). To provide GPs with some structure during the ‘person-centered consultation’, this model is suggested. During the consultation, a goal attainment (GA) procedure will be applied to evaluate and monitor the person-centered care process. GA can be described as a way of scoring the extent to which patients achieve their individual goals throughout intervention (Turner-Stokes, 2009). During the first ‘person-centered consultation’, goals, outcome measures and time period till measurement are formulated. In the follow-up consultations, goals attainment will be discussed. In addition, ongoing person-centered management is addressed in follow-up consultations with duration till follow-up according to the circumstances of the patients. Moreover, the shared goal setting and decision-making will indicate whether other disciplines (eg, practice nurses (PNs), physiotherapists and psychologists) will be involved in the care process.

#### Test phase

During the test phase, a larger group of general practices will adopt the developed approach. A longitudinal nested case-cohort design will be applied in order to explore the impact of the developed approach for chronic care on the ‘Quadruple aim’ outcome. Patients are asked to complete questionnaires to collect information about their demographics, health literacy, chronic conditions, functional ability, health care utilization, quality of life, their experiences with received primary care and sickness leave (Table 1). Moreover, to evaluate quality of the care, information from all patients eligible for the study will be obtained from GPs electronic patient records. Data will be collected at baseline (T0) and every six months for 24 months.

**Table 1.** Measures of COPILOT-study

	Concept	Instrument	0*	12	24
Patient					
*Primary outcomes*	patients’ experience of health care delivery (1)	PACIC	X	X	X
	patients’ experience of health care delivery (1)	NPS	X	X	X
	patients’ experience with integration of care (1)	QUOTE	X	X	X
	Quality of life, generic health (2)	EQ-5D-5L	X	X	X
	Quality of life, social perspective (2)	ASCOT	X	X	X
	Impact of self-reported disease-related limitations (2)	Developed questions	X	X	X
	Clinical indicators of disease severity and activity (2)	GPs electronic patient records	X		X
	Resource use & sickness leave (4)	Tic-P	X	X	X
*Other variables*	Age		X	X	X
	Gender		X	X	X
	Social economic status		X	X	X
	*n* chronic diseases		X	X	X
	Type chronic diseases		X	X	X
	Health literacy	HLS-EU-Q16	X	X	X
	Functional and social ability	InterRAI CU-SR	X	X	X
Primary care professional					
*Primary outcomes*	Job satisfaction (3)	Developed questionnaire	X		X

PACIC = patient assessment of chronic illness care; NPS = net promotor score; QUOTE = quality of care through patients’ eyes; EQ-5D-5L = EuroQol 5D – 5 level version; ASCOT = adult social care outcomes toolkit; CU-SR = check-up self-report; Tic-P = trimbos and iMT questionnaire on costs associated with psychiatric illness; HLS-EU-Q16 = 16-item Dutch version of the European health literacy short survey questionnaire; VAS = visual analogue scale.

* Months after baseline.

(1) Patient experience with chronic primary care.

(2) Patients’ health.

(3) Societal costs and costs of resources use.

(4) Care professionals’ experiences with management of multimorbidity.

### Recruitment

#### General practices

During the first research cycle, GPs and their PNs from 13 general practices will be recruited. These general practices are affiliated with a primary care cooperation named ‘Huisartsen Coöperatie Zuid-Kennemerland’ (HCZK). During the test phase, the developed person-centered care approach will become available for all general practices affiliated with HCZK (87 practices); all 125 GPs will be invited to adopt the developed approach.

### In- and exclusion criteria

In- and exclusion criteria are described in Box 1.

Box 1 In-and exclusion criteria for selecting care professionals and patients

Inclusion criteria

Eligible general practitioners & practice nurses

General practitoners and practice nurses from general practices affiliated with HCZK.

*Eligible patients*

- ≥3 diagnoses* from the list ‘Chronic conditions’ (Table 2)**
- Mentally competent***
- Aged 18 or older

Exclusion criteria

- Terminally ill
- Mentally handicapped (ICPC-code P85)
- Diagnosed with dementia (ICPC-code P70)
- Severely hearing or visual impaired (ICPC-code: H86, F94)
- Insufficient command of the Dutch language

*The criterion ≥ 3 chronic diagnoses was formulated after considering estimations of eligible patients based on routine care records of three local practices who are part of Academisch Netwerk Huisartsengeneeskunde (ANH). Consequently, a manageable amount of patients to participate in this study was achieved. Moreover, since this criterion was applied in an earlier study in a comparable setting on this topic (Salisbury, 2018), we chose to stay in line with existing literature and therefore formulated this criterion.

**This list ‘chronic diseases’ is developed via multiple brainstorm sessions with the group of initiating GPs and includes chronic conditions which are considered as ‘in need of chronic primary care’. Conditions are coded using the International Classification of Primary Care (ICPC); Supplementary Material. Consensus was reached after comparing the developed list with existing lists of chronic conditions (O’Halloran, Miller and Britt, 2004).

***Mental competence is defined as patients without dementia and patients who are not mentally handicapped. Patients with mild cognitive impairment will not be excluded.

**Table 2.** Chronic conditions

*Clusters*	Diseases
*HIV/Aids*	HIV/Aids
*Cancer*	All malignant cancer types
*Bowel disorders*	Diverticular disease
	Crohn’s disease
	Ulcerative colitis
*Cardiovascular*	Congenital heart disease
	Infectious disease of heart and/or blood vessels
	Acute rheumatoid heart disease
	Non-rheumatic valvular Heart Disease
	Heart failure
	Angina pectoris
	Acute myocardial infarction
	Atrial fibrillation/flutter
	Hypertension
	Transient ischemic attack (TIA)
	Cerebrovascular accident (CVA)
	Intermittent claudication
	Aneurysm aortae
	Hypercholesterolemia
*Musculoskeletal*	Fibromyalgia
	Rheumatoid arthritis
	Cox arthrosis
	Gon arthrosis
	Other arthrosis
	Cervical spine syndromes
	Osteoarthritis spondylosis of the spine
	Low back pain with radiation
	Osteoporosis
*Neurologic*	Multiple sclerosis (MS)
	Parkinson’s disease
	Epilepsy
	Migraine
	Cluster headache
	Trigeminal neuralgia
	Other neuropathies
*Alcohol abuse*	Chronic alcohol abuse
*Psychiatric*	Sleeping disorder
	Schizophrenia
	Affective psychosis
	Depression
	Anxiety disorder
	Personality disorder
*Respiratory*	Chronic obstructive pulmonary disease (COPD)
	Asthma
	Chronic bronchitis
*Thyroid*	Persistent thyroglossal duct/cyst
	Benign neoplasms of thyroid gland
	Hyperthyroidism
	Hypothyroidism
*Diabetes mellitus*	Diabetes mellitus type I
	Diabetes mellitus type II
*Urinary*	Kidney disease
*Psoriasis*	Psoriasis with methotrexate use
*Obesity*	Adiposity
*Smoking*	Tobacco abuse
*Eye disease*	Macular degeneration

#### Patients

Patients will be recruited from general practices affiliated with HCZK.

Different (sub)groups can be identified:

1. All patients eligible for the study. This group will be asked to participate in the study and to fill in a questionnaire at T0.
2. Patients from group 1 who filled in the questionnaire at T0 but do not want to participate in the study or receive follow-up evaluations.
3. Patients from group 1 who receive care via the new chronic care approach.
4. Patients from group 1 and 3 who are willing to participate in the study and its follow-up evaluations.
5. Patients from group 3 who are willing to participate in a semi-structured interview or focus groups to share their experiences with the new chronic care approach.

#### Patient selection procedure

Patients will be chosen from a list of eligible patients extracted from the GPs electronic database based on the defined in- and exclusion criteria. Between research cycles, the list of eligible patients will be updated. GPs have to make their considerations explicit to the researcher so this can be taken into account during the analyses. To encourage variety in type of patients, GPs are asked to invite at least one patient from the following five predefined substrata: patients (i) who have at least one chronic condition within the structured disease management programs, (ii) who have none of the diseases within the structured disease management programs, (iii) with low care needs (less than 20 contacts in the last two years), (iv) with medium care needs (20–40 contacts in the last two years) and (v) patients with high care needs (more than 40 contacts in the last two years) (Hameleers *et al*., 2017).

### Measurement instruments

Variables measured in this study are summarized in Table 1. These will be assessed using a combination of self-report instruments and by data from GPs electronic patient records.

#### Test phase: primary outcomes

Primary outcomes concern the ‘Quadruple aim’ outcomes, consisting of experiences of patients and professionals, patient outcomes and costs of general practice care.

1. ***Patient* experience with chronic primary care**

This so-called patient-reported experience measures will be assessed with (i) Patient Assessment of Chronic Illness Care (PACIC) measure (Glasgow *et al*., 2005), (ii) the Net promotor score (NPS) measure (Hamilton *et al*., 2014) and (iii) one statement out of the QUality Of care Through patients’ Eyes (QUOTE) measure (Sixma *et al*., 2000). The statement ‘the GP I have seen during the past year always communicates with other health and social care providers about health services I require’ was added in order to evaluate patients’ experiences with integration of care between primary and secondary health care providers. In our opinion, this aspect of care delivery is lacking in the aforementioned measures.

2. ***Patients outcomes***
a. **Patient-reported outcomes**

Quality of life

Quality of life will be assessed with a generic health-related perspective by the EuroQol 5D – 5 level version (EQ-5D-5L) measure (Herdman *et al*., 2011; Versteegh *et al*., 2016) and with a social perspective with the adult social care outcomes toolkit (ASCOT) measure (Malley *et al*., 2012; van Leeuwen *et al*., 2015), which are so-called ‘patient-reported outcome measures’ (PROMS).

*Impact of self-reported disease-related limitations*

Impact of self-reported limitations related to diseases will be measured with the visual analogue scale (VAS). The VAS provides a continues scale for magnitude estimation ranging from 0 to 10 (Carlsson, 1983: 87).

b. **Clinical outcomes**

Clinical indicators of disease severity and activity

To evaluate quality of care of the highly frequent chronic conditions: diabetes mellitus, COPD and CVRM (afore managed via structured disease management programs), indicators collected from the GPs electronic database will be analyzed. Indicators include lab results, for example, HbA1c, clinical measures, for example, blood pressure, prescribed medication and contact frequency.

3. ***Care professionals’ experiences with management of multimorbidity***

The researchers developed in close collaboration with the initiating GPs a topic list concerning job satisfaction and experienced regulatory burden. Based on these topics, statements were formulated. Response options range from 0 (agree) to 10 (totally disagree). The questionnaire was pilot, tested and adapted.

4. ***Societal costs and costs of resources use***

*Resource use*

Health care utilization will be assessed using items from the Trimbos and iMTA questionnaire on Costs associated with Psychiatric Illness (Tic-P). The Tic-P is an instrument that assesses self-reported health care utilization, medication use, informal care, absenteeism from paid and unpaid work, and presenteeism (Hakkaart-van Roijen, 2002). Since this questionnaire was developed for psychiatric care, we made some adjustments to be applicable to primary care.

*Sickness leave*

Sickness leave will be assessed with a selection of adjusted questions out of the above-mentioned Tic-P measure (Hakkaart-van Roijen, 2002).

#### Other variables

Patient characteristics, including, age, gender, marital status, household, education, Functional and social ability and health literacy will be derived from self-report measures. Age and gender will also be derived from GPs electronic patient records.

World Health Organization (WHO, 1998) defined health literacy (HL) as ‘the cognitive and social skills which determine the motivation and ability of individuals to gain access to, understand and use information in ways which promote and maintain good health’. We included the 16-item Dutch version of the European Health Literacy short Survey questionnaire (HLS-EU-Q16), which is a good approximation of the full 47-item version. It has a high correlation (*r* = 0.82) between the HLS-EU-47 score and the HLS-EU-Q16 score and a 75.8% agreeing classification of respondents: insufficient, limited and sufficient health literacy. The sixteen items are formulated as questions and rated on a four-point Likert scale ranging from ‘very easy’ to very difficult’. The HL scores are calculated according to the recommendations of the European Health Literacy Project. Scores of 0–8 are considered as insufficient HL, scores between 9 and 12 as limited HL, and scores of 13 or more as sufficient HL (Vandenbosch, 2016).

Patients’ functional and social abilities are assessed in comprehensive way by the InterRAI Check-Up (CU-SR) self-report assessment. InterRAI CU-SR consists of almost 90 items concerning multiple domains, such as cognitive status and communication skills, experienced well-being, daily activities, health conditions, disease diagnoses, nutrition and finances and stressors. Internationally validated scales are embedded in the Check-Up (InterRAI, 2018).

### Sample size

We estimate that 56 patients per 1000 per general practice are eligible based on routine care records of 3 local practices who are part of the ‘Academisch Netwerk Huisartsengeneeskunde’ (ANH) affiliated with VU University Medical Center (VUmc). The participating GPs invite between 4 and 10 patients per research cycle.

### Data analysis

#### Qualitative analysis: focus groups and semi-structured interviews

Recorded focus groups and semi-structured interviews will be analyzed by two researchers and categories will be formulated. Throughout ongoing discussion between the researchers, categories will be reduced into major themes. Based on these themes (if needed), the approach for chronic primary care will be adjusted.

#### Quantitative analysis

We will use descriptive statistics for the demographic features of the cohort.

*Quadruple aims*

To explore the impact of the new approach for chronic primary care on the ‘Quadruple aim’ outcomes, a mixed linear regression model will be applied. Participants who underwent the new approach (‘cases’) will be compared to those who did not (‘controls’), adjusting for (eg, demographic, social, functional and medical) differences. In addition, confounding and effect modification will be explored. The four ‘Quadruple aim’ outcomes will be analyzed both separately and in conjunction. For the latter, we aim to use a multi-attribute value-based method or multi-criteria decision analysis (MCDA), which applies a weighing of the four ‘Quadruple aim’ outcomes to calculate an overall value score (Rutten-van Mölken, 2018). Moreover, predefined subgroups, for example, age groups, care groups (low, medium and high care, and social economic status groups) will be analyzed.

#### Process analysis

To evaluate the development phase of this study, a process analysis will be conducted in which we identify facilitating and impeding factors in the various cycles and from multiple perspectives (professionals, patients and insurers).

## Discussion

To our knowledge, this is the first study which aims to develop a proactive person-centered primary care approach for patients with multimorbidity in a bottom-up manner by applying action-based research design. Previous research shows that capturing the effectiveness of innovative care models for patients with multiple chronic conditions in general practice is challenging. For example, Salisbury *et al*. conducted a clustered RCT in 33 general practices in which they compared patient-centered care with usual care (Salisbury *et al*., 2018). Their results showed that, although the intervention was effective in improving the patient’s experience of patient-centered care, it was not associated with benefits in quality of life or the burden of illness or treatment. Our study builds upon these results and will additionally assess more personalized outcomes and the impact on GPs and PNs, in an action-based approach, in which involvement of primary care professionals in the decision-making process creates the opportunity to develop together in a stepwise fashion a new approach for chronic care which is the best fit for clinical practice.

Besides strengths, we also recognize limitations and potential risks. First, by design, during the development phase GPs choose the patients to put into practice the new approach for chronic primary care. In addition, since the list of chronic conditions includes 56 conditions (Table 2), we expect great variance in types of patients who undergo the new chronic primary care approach. Consequently, the new model may not be suitable for all types of patients. Even so, we hope this study will help us identify subgroups of multimorbid patients who will benefit most from the new chronic primary care approach. Second, a potential risk is that GPs are unable to recruit enough patients to undergo the new primary chronic care approach with. The success of this study depends highly upon the actions of the GPs. Even though the GPs initiated this project and there is a financial compensation, time constraints might hamper the performance of the ‘broad chronic consultations’. To avoid time management problems, GPs are encouraged to invite and schedule all 4–10 patients at the beginning of the ‘action phase’. Third, in terms of data collection, burden of data collection on patients as well as practices is considered. Regarding the burden on patients, online questionnaires with multiple skip rules were developed, piloted and evaluated as user-friendly and understandable. With regard to the burden of data collection on practices, we supported the GPs and their coworkers in several ways, for example, by facilitating each participating GP with a search query to extract a list of eligible patients based on their electronic patient records to avoid time-consuming searches. Another potential risk is that during the development phase, the saturation is not met after three research cycles. As a result, the development phase will continue on and the test phase will be extended. Furthermore, future practices may differ from pilot practices that warrant further adaptation of the newly developed chronic care approach. However, we expect the pilot practices to already reflect a rather diverse group within the cooperative (HCZK). Moreover, the aim of this study is to develop a new approach instead of a new rigor protocol. This new approach leaves room for tailor-made adjustments regarding the needs of the practices.

Sustainability will be addressed in several ways. First, the development of a new approach for chronic care was initiated by GPs themselves and gained widespread support. For example, Dutch GPs participating in societal change interest groups, health insurers, regulatory authorities and the government emphasize the need to reorganize in order to provide more room for professionals to add value by more personalized care in addition to applying disease management protocols (Landelijke Huisartsen Vereniging (LHV), 2018). Furthermore, the use of an action-based research design increases involvement and chances to be sustainable. Together with the Regional Cooperative (HCZK), we aim to produce implementation guidance that can support new practices to adopt the new way of working. The regional cooperative can also offer support with trainings. Moreover, in collaboration with the involved health insurer, we aim to describe resource needs to establish this person-centered, non-disease-specific care approach in order to encourage implementation.

Since this study is explorative, no strong conclusion can be drawn upon the impact of the new approach for chronic primary care on the ‘Quadruple aim’ outcomes. Nonetheless, this study will contribute to enhance our knowledge on management of chronic care in primary care setting for patients with multimorbidity. If successful, future research could evaluate its ‘Quadruple aim’ effectiveness on the short and long term, for example, in an RCT.

## Supplementary material

For supplementary material accompanying this paper visit https://doi.org/10.1017/S1463423619000550. click here to view supplementary material
