# Supplementary material for: From protocolized to person-centered chronic care in general practice: study protocol of an action-based research project (COPILOT)
Source: Prim Health Care Res Dev. 2019 Sep 24;20:e134. doi: 10.1017/S1463423619000550 (PMC6764186; doi:10.1017/S1463423619000550)
Supplement: Supplementary file 1 [file S1463423619000550sup001.docx]

| **Table 2Chronic conditions** | |  |
| --- | --- | --- |
| **Clusters** | **Diseases** | **ICPC-code(s)** |
| HIV/Aids | HIV/Aids | B90 |
| Cancer | All malignant cancer types | A79, B72-B72.01-B72.02 , B73, D74, D75, D76, D77-77.01- D77.02-D77.03-D77.04, L71-L71.01, N74, R84, R85, S77-S77.01-S77.02-S77.03-S77.04, T71, U75, U76, U77, W72, X75, X76-X76.01, X77-X77.01-X77.02, Y77, Y78-Y78.01-Y78.02-Y78.03 |
| Bowel disorders | Diverticular disease | D92 |
|  | Crohn disease | D94 |
|  | Ulcerative colitis | D94 |
| Cardiovascular | Congenital heart disease | K73-K73.01-K73.02 |
|  | Infectious disease of heart and/or blood vessels | K70 |
|  | Acute rheumatoid heart disease | K71-K71.01-K71.02 |
|  | Non-rheumatic Valvular Heart Disease | K83-K83.01-K83.02 |
|  | Heart failure | K77-K77.01-K77.02 |
|  | Angina Pectoris | K74-K74.01-K74.02 |
|  | Acute myocardial infarction | K75 |
|  | Atrial fibrillation/flutter | K78 |
|  | Hypertension | K86, K87 |
|  | Transient Ischemic Attack (TIA) | K89 |
|  | Cerebrovascular accident (CVA) | K90 |
|  | Intermittent claudication | K92 |
|  | Aneurysm aortae | K99 |
|  | Hypercholesterolemia | T93 |
| Musculoskeletal | Fibromyalgia | L18, L18.01 |
|  | Rheumatoid arthritis | L88-L88.01-L88.02 |
|  | Coxarthrosis | L89 |
|  | Gonarthrosis | L90 |
|  | Other arthrosis | L91 |
|  | Cervical spine syndromes | L83-L83.01 |
|  | Osteoarthritis spondylosis of the spine | L84-L84.01-L84.02 |
|  | Low back pain with radiation | L86-L86.01 |
|  | Osteoporosis | L95-L95.02-L95.02 |
| Neurologic | Multiple sclerosis (MS) | N86 |
|  | Parkinson's disease | N87-N87.01 |
|  | Epilepsy | N88 |
|  | Migraine | N89 |
|  | Cluster headache | N90 |
|  | Trigeminal neuralgia | N92 |
|  | Other neuropathies | N94 |
| Alcohol abuse | Chronic alcohol abuse | P15 |
| Psychiatric | Sleeping disorder | P06 |
|  | Schizophrenia | P72 |
|  | Affective psychosis | P73-P73.02 |
|  | Depression | P76-P76.01 |
|  | Anxiety disorder | P74-P74.01-P74.02 |
|  | Personality disorder | P80-P80.01-P80.02 |
| Respiratory | Chronic Obstructive Pulmonary Disease (COPD) | R95 |
|  | Asthma | R96-R69.01-R69.02 |
|  | Chronic bronchitis | R91-R91.01-R91.02 |
| Thyroid | Persistent thyroglossal duct/cyst | T78 |
|  | Benign neoplasms of thyroid gland | T72 |
|  | Hyperthyroidism | T85 |
|  | Hypothyroidism | T86 |
| Diabetes Mellitus | Diabetes Mellitus type I | T90.01 |
|  | Diabetes Mellitus type II | T90.02 |
| Urinary | Kidney disease | U99 |
| Psoriasis | Psoriasis with methotrexate use | S91 |
| Obesity | Adiposity | T83 |
| Smoking | Tobacco abuse | P17 |
| Eye disease | Macular degeneration | F84 |
